# ST-Segment Elevation Acute Myocardial Infarction Complicated by Cardiogenic Shock: Early Predictors of Very Long-Term Mortality

**DOI:** 10.3390/jcm10112237

**Published:** 2021-05-21

**Authors:** Nicola Cosentino, Marta L. Resta, Alberto Somaschini, Jeness Campodonico, Giampaolo D’Aleo, Giovanni Di Stefano, Claudia Lucci, Marco Moltrasio, Alice Bonomi, Stefano Cornara, Andrea Demarchi, Gaetano De Ferrari, Antonio L. Bartorelli, Giancarlo Marenzi

**Affiliations:** 1 Centro Cardiologico Monzino IRCCS, 20138 Milan, Italy; resta.martal@gmail.com (M.L.R.); jeness.Campodonico@cardiologicomonzino.i (J.C.); giampaolodaleo@gmail.com (G.D.); giovannidistefano91@gmail.com (G.D.S.); claudia.lucci@ccfm.it (C.L.); marco.moltrasio@ccfm.it (M.M.); alice.bonomi@ccfm.it (A.B.); Antonio.Bartorelli@ccfm.it (A.L.B.); giancarlo.marenzi@ccfm.it (G.M.); 2 Coronary Care Unit and Laboratory of Clinical and Experimental Cardiology—Fondazione IRCCS Policlinico San Matteo, 27100 Pavia, Italy; alberto.somaschini08@gmail.com (A.S.); stefano.cornara@gmail.com (S.C.); andrea.demarchi02@universitadipavia.it (A.D.); 3 Unit of Cardiology, Department of Molecular Medicine, Università degli studi di Pavia, 271000 Pavia, Italy; 4 Dipartimento di Scienze Mediche, Cardiologia Città della Salute e della Scienza, Università di Torino, 10126 Torino, Italy; gaetanomaria.deferrari@unito.it; 5 Department of Biomedical and Clinical Sciences “Luigi Sacco”, University of Milan, 20157 Milan, Italy

**Keywords:** cardiogenic shock, ST-elevation myocardial infarction, primary percutaneous coronary intervention, long-term mortality, risk score

## Abstract

Background. Cardiogenic shock (CS) is the leading cause of in-hospital mortality in ST-segment elevation myocardial infarction (STEMI). Only limited data are available on the long-term outcome of STEMI patients with CS undergoing contemporary treatment. We aimed to investigate long-term mortality and its predictors in STEMI patients with CS and to develop a risk score for long-term mortality prediction. Methods and Results. We retrospectively included 465 patients with STEMI complicated by CS and treated with primary angioplasty and intra-aortic balloon pump between 2005 and 2018. Long-term mortality, including both in-hospital mortality and all-cause mortality following discharge from the index hospitalization, was the primary endpoint. The long-term mortality (median follow-up 4 (2.0–5.2) years) was 60%, including in-hospital mortality (34%). At multivariate analysis, independent predictors of long-term mortality were age (HR 1.41, each 10-year increase), admission left ventricular ejection fraction (HR 1.51, each 10%-unit decrease) and creatinine (HR 1.28, each mg/dl increase), and acute kidney injury (HR 1.81). When these predictors were pooled together, the area under the curve (AUC) for long-term mortality was 0.80 (95% CI 0.75–0.84). Using the four variables, we developed a risk score with a mean (cross-validation analysis) AUC of 0.79. When the score was applied to in-hospital mortality, its AUC was 0.79, and 0.76 when the score was applied to all-cause mortality following discharge. Conclusions. In STEMI patients with CS, the risk of death is still substantial in the years following the index event. A simple clinical score at the time of the index event accurately predicts long-term mortality risk.

## 1. Introduction

Cardiogenic shock (CS) is the leading cause of in-hospital mortality in patients with ST-segment elevation myocardial infarction (STEMI). It occurs in approximately 7–10% of patients, mainly within the first hours after symptom onset [1,2,3]. The usual treatment is primary percutaneous coronary intervention (pPCI) and peri-procedural circulatory support with an intra-aortic balloon pump (IABP) [4,5]. Despite this therapeutic approach, early mortality of these patients is still high, ranging from 30% to 50% [4,5,6,7,8,9,10]. The prognostic impact of CS on long-term mortality has been scantily investigated, and controversial results have been provided thus far. On the one hand, some studies have reported that CS predicts long-term mortality in STEMI patients who survived the index hospital stay [11,12]. On the other hand, other studies failed to find such an association [13,14]. Different CS definition, heterogeneous population and therapeutic approach, as well as different length of follow-up, may explain, at least in part, the conflicting data [11,12,13,14]. When studies focusing only on patients with acute myocardial infarction and CS are considered, a very high long-term mortality rate—up to almost 70% at six years—has been reported in large randomized trials [15,16]. This suggests that the risk of death is still substantial even after the acute phase. Thus, early recognition of the clinical characteristics associated with long-term mortality risk of STEMI patients with CS is an important target that may help to better determine where to concentrate major efforts in terms of need for temporary and durable mechanical circulatory support. Indeed, as the temporal trend of prevalence of CS in patients with STEMI has been increasing [17], better and earlier selection of CS patients to refer for advanced heart failure treatments will be the next focus to further reduce their mortality rate.

In this study, we investigated very long-term mortality, including in-hospital mortality, in a real-world population of consecutive patients with STEMI complicated by CS at hospital admission who were treated with pPCI and IABP. Moreover, we developed a simple, easy-to-use and readily available risk score for the early prediction of overall mortality in this clinical setting.

## 2. Methods

### 2.1. Study Population

The data analyzed in this retrospective study were obtained from consecutive patients with STEMI complicated by CS at hospital admission who were treated with pPCI and IABP at the Centro Cardiologico Monzino in Milan, Italy, between 1 January 2005 and 1 January 2018, and the Policlinico San Matteo of Pavia, Italy, between 1 January 2005 and 25 September 2017. Patients were included if they presented within 24 h from symptom onset and with CS (persistent systemic hypotension and signs of impaired organ perfusion caused by severe left ventricular dysfunction, right ventricular infarction, or mechanical complications of infarction, and not due to hypovolemia, hemorrhage, bradyarrhythmias, or tachyarrhythmias). Patients undergoing cardiac transplantation or left ventricular assist device implantation during index hospitalization were excluded. The study was approved by the Ethics Committee.

### 2.2. Study Protocol

In all patients, IABP was started in the Catheterization Laboratory at a frequency of 1:1 before or soon after pPCI. The use of inotropic and vasoactive agents, diuretics, and the indication for endotracheal intubation and mechanical ventilator support was left to the discretion of the Coronary Care Unit (CCU) cardiologists based on standards of care. Primary PCI was performed by a 24-h on-call interventional team according to standard clinical practice. Standard guide catheters (6Fr), guide wires, balloon catheters, and coronary stents were used via radial or femoral approach. Pharmacological therapy and post-stenting antithrombotic treatment were administered according to institutional protocols and guideline recommendations. The mode of revascularization (pPCI with treatment of the target lesion only, PCI of the target lesion, plus additional immediate or staged PCI of non-target lesions) was left to the discretion of the operator.

Demographic, clinical, biochemical, and echocardiographic data were obtained from all patients. An echocardiogram was performed in all patients within the first hours of hospital admission. Left ventricular ejection fraction (LVEF) was calculated by the Simpson’s rule. Serum creatinine concentration was measured by means of the Jaffe method, at hospital admission (before pPCI) and every day during CCU stay. Acute kidney injury (AKI) was defined as an increase in serum creatinine ≥0.5 mg/dl during the first 72 h of hospital admission [18]. For each patient, we calculated the maximum contrast dose (MCD) by using the formula proposed by Cigarroa et al.: MCD (mL) = (5 × body weight (kg)) divided by serum creatinine (mg/dL). From this contrast limit, we determined the contrast ratio by dividing the contrast amount administered during pPCI by the calculated MCD [19].

The primary endpoint of the study was long-term mortality, including both in-hospital mortality and all-cause mortality following discharge from the index hospitalization. Secondary endpoints of the study were in-hospital mortality and all-cause mortality following discharge from the index hospitalization, considered separately. Patient follow-up was performed by telephone calls or by retrieving data from administrative registries by dedicated medical personnel.

### 2.3. Statistical Analysis

Continuous variables are presented as mean ± SD, and were compared using the *t*-test for independent samples. Non-normally distributed variables are presented as median and interquartile ranges and were compared with the Wilcoxon rank-sum test. Categorical data were compared using the chi-square test or Fisher’s exact test, as appropriate. A multivariable Cox model regression was developed to identify independent predictors of long-term mortality, selected among variables identified at stepwise analysis. All the variables reported in Table 1 were initially considered at stepwise analysis. Results are presented as hazard ratio (HR) and 95% confidence intervals (CI) and adjusted for year of enrollment.

The ability of these variables, considered separately and in combination, to predict long-term mortality was quantified by the area under the receiver-operating characteristic (ROC) curve (AUC).

A risk score for the primary endpoint was then developed. A logistic regression model was employed, including all the independent predictors of the primary endpoint. The risk score (predicted probability of event at 4 years) was computed for each patient using the following formula [20]:

e^(*β*_0_+∑*β_i_X_i_*)^ (1)

predicted probability of long-term mortality =

1 + e^(*β*_0_+∑*β_i_X_i_*)^ (2)

where *β*_0_ is the constant of the logistic regression equation, and *β_i_* is the coefficient of the variable *X_i_* in the logistic regression equation. The 4-year time interval was chosen as it represents the median follow-up of the whole study population. A cross-validation procedure was employed to calculate the coefficients. The study sample was randomly split in half 200 times, the coefficients of the risk score were estimated in the first arm (training set), and its AUC was subsequently tested in the second half (testing set). The mean value of each coefficient was considered for the final score. Calibration of the score was evaluated by dividing the sample in deciles of risk and by comparing the observed events with the predicted events in each decile (Hosmer-Lemeshow test).

All tests were 2-tailed, and *p* < 0.05 was required for statistical significance. All analyses were performed using SAS version 9.4 (SAS Institute, Cary, NC, USA).

## 3. Results

We enrolled 477 STEMI patients with CS at hospital admission. Twelve patients were lost to follow-up. Thus, the final analysis included 465 patients (mean age 68 ± 12 years; 338 men).

### 3.1. Primary Endpoint

**Figure 1:**
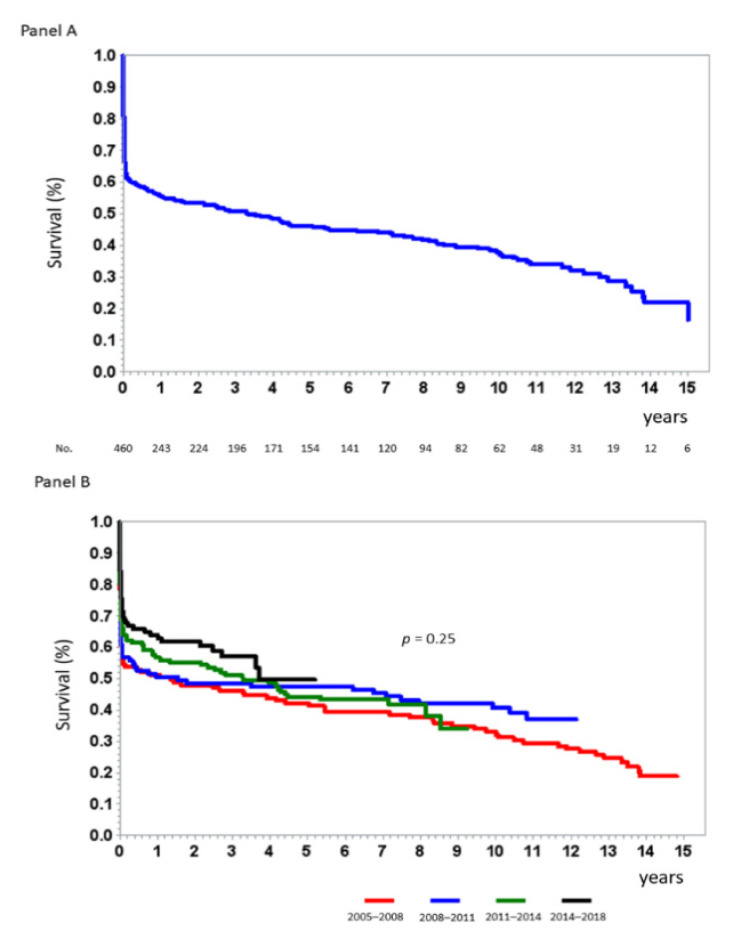
Kaplan-Meier curves showing the long-term survival rate of the ST-segment elevation acute myocardial infarction patients with cardiogenic shock enrolled in the study (Panel **A**) and stratified according to quartiles of date of index hospitalization (Panel **B**). *p* value by Log rank test.

The median long-term follow-up was 4 (2.0–5.2) years. The cumulative mortality of the study population was 60% (*n* = 281) (Figure 1).

The baseline clinical characteristics of patients surviving and of those non-surviving during the entire study period are reported in Table 1. Non-surviving patients were older and more likely to have lower LVEF and higher admission serum creatinine and glucose. The two groups were similar in terms of cardiovascular risk factors, prior cardiovascular events, time-to-reperfusion and enzymatic peak (creatine kinase-MB isoenzyme: 305 ± 222 ng/mL and 315 ± 258 ng/mL in surviving and non-surviving patients, respectively; *p* = 0.67). Contrast volume was also similar in the two groups, while contrast ratio was significantly higher in non-surviving patients.

At multivariate Cox regression analysis with stepwise selection, the following variables remained significant independent correlates of long-term mortality: age (HR 1.41, 95% CI 1.27–1.92; *p* < 0.0001 for each 10-year increase), LVEF (HR 1.51, 95% CI 1.31–1.73; *p* < 0.0001 for each 10% unit decrease), serum creatinine concentration (HR 1.28, 95% CI 1.10–1.50; *p* < 0.0001, for each mg/dl increase), and AKI occurrence during the index hospitalization (HR 1.81, 95% CI 1.35–2.41; *p* < 0.0001).

**Figure 2:**
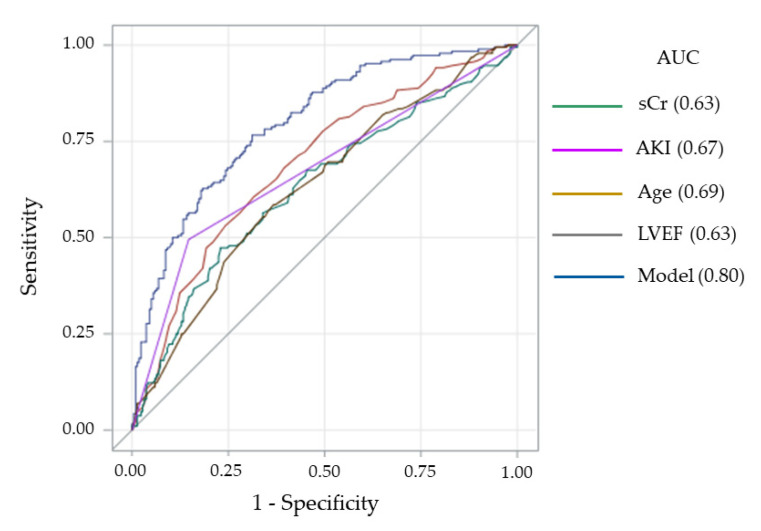
Receiving operating characteristic curves and corresponding area under the curves (AUC) for long-term mortality of each independent predictor considered separately and in combination (Model). AKI = acute kidney injury; LVEF = left ventricular ejection fraction; sCr = serum creatinine concentration.

Figure 2 shows the AUC of each independent predictor of cumulative long-term mortality considered separately and in combination (Model). When these predictors were pooled together, the predictive accuracy of the model improved significantly with an AUC of 0.80 (95% CI 0.75–0.84; *p* < 0.0001).

Using the four variables as risk indicators for long-term mortality, we developed a risk score (Table 2).

**Figure 3:**
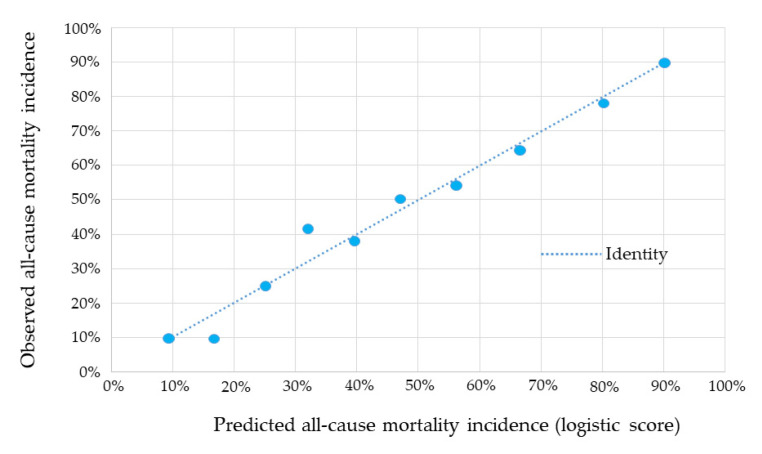
Concordance between the long-term mortality incidence predicted by the score and that observed in the entire population, stratified by deciles of risk.

Cross-validation analysis showed high reproducibility of the score, with a mean AUC for long-term mortality of 0.79 (95% CI 0.72–0.85; *p* < 0.0001). The concordance between the long-term mortality predicted by the score and that observed in the entire population, stratified by deciles of risk, is reported in Figure 3.

### 3.2. Secondary Endpoints

**Figure 4:**
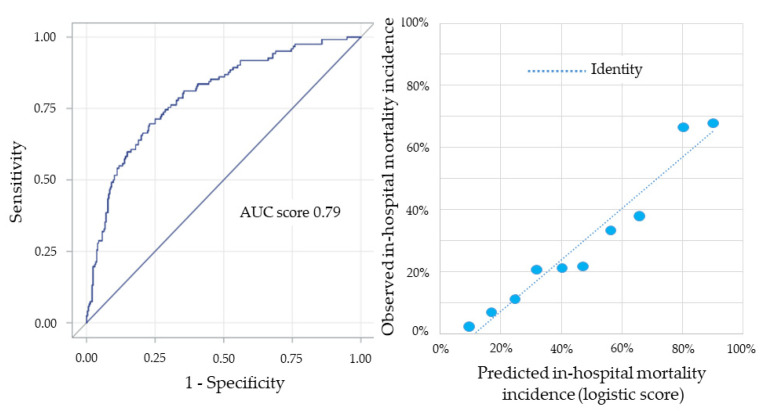
Left panel: receiving operating characteristic curve and corresponding area under the curve (AUC) for in-hospital mortality prediction of the risk score. Right panel: concordance between the in-hospital mortality incidence predicted by the score and that observed in the entire population, stratified by deciles of risk.

In-hospital mortality of our study population was 34% (*n* = 160). When also patients discharged from the index hospitalization were considered (*n* = 305), the median long-term follow-up was 6.9 (5.2–11.7) years. The mortality rate of discharged patients was 40% (*n* = 121), with a yearly mortality rate of 7%. When the score developed for the primary endpoint was applied to in-hospital mortality, its AUC was 0.79 (95% CI 0.74–0.83; *p* < 0.0001) (Figure 4, left panel).

**Figure 5:**
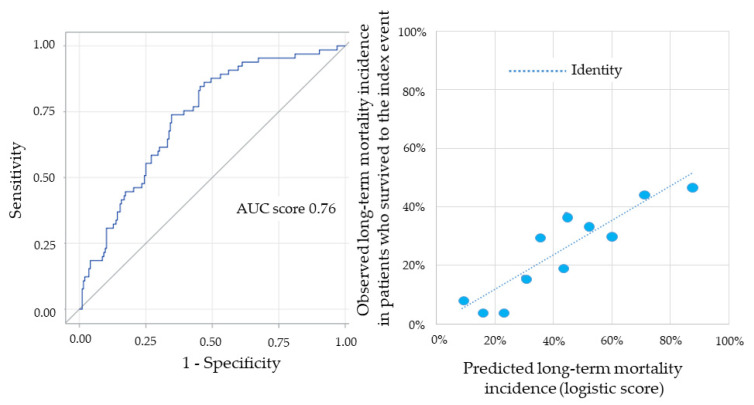
Left panel: receiving operating characteristic curve and corresponding area under the curve (AUC) for the risk score prediction of all-cause mortality following discharge from the index hospitalization. Right panel: concordance between the incidence of all-cause mortality following discharge from the index hospitalization predicted by the score and that observed in the entire population, stratified by deciles of risk.

The concordance between predicted and observed in-hospital mortality stratified by deciles of risk, according to the risk score, is shown in Figure 4 (right panel). The AUC of the score applied to all-cause mortality following discharge from the index hospitalization was 0.76 (95% CI 0.71–0.81; *p* < 0.0001) (Figure 5, left panel). The concordance between predicted and observed mortality in these patients stratified by deciles of risk, according to the risk score, is shown in Figure 5 (right panel).

## 4. Discussion

The main finding of this study is that the long-term mortality of STEMI patients with CS remains high after hospital discharge, with an additional absolute mortality increase of 26% at long-term follow-up (7% per year) compared with a 34% in-hospital mortality. A simple clinical score calculated during the index event can accurately predict the overall mortality risk.

Despite the progressive decline in mortality rates in CS, largely attributed to advances in early revascularization strategies, including the increased availability and improved safety profile of pPCI, it remains the most common cause of death in STEMI patients [4,5]. Robust evidence has been provided regarding the early mortality risk associated with STEMI complicated by CS [21,22], with some scoring systems showing good predictive value regarding short-term mortality [23,24,25]. Indeed, accurate risk stratification is critical in this acute setting in order to guide treatment decisions. However, the long-term mortality of patients with acute myocardial infarction and CS has been less widely investigated (Table 3) and non-homogeneous data have been provided.

On the one hand, Spyridopoulos et al. [13] reported that CS is not able to predict mortality in STEMI patients who survived the index hospital stay. On the other hand, Hemradj et al. [11] found that CS was associated with an almost three-fold higher long-term mortality adjusted risk in STEMI patients surviving the acute event. Moreover, data from three nationwide French registries reported a stepwise decline in 30-day mortality from 70% in 1995 to 63% in 2000 and to 51% in 2005, while mortality from one month to one year remained high (>20%) and did not improve over time [22]. However, these studies considered a one-year follow-up. Only few studies focused on longer follow-up and, again, provided conflicting results. In the Coronary Revascularization Demonstrating Outcome study in Kyoto Acute Myocardial Infarction (CREDO-Kyoto AMI) registry, CS was a robust predictor of six-month mortality, but failed to predict mortality at longer (up to seven years) follow-up in 3942 STEMI patients treated with pPCI (14). Conversely, Hosseiny Doost et al. found a close association between CS and three-year mortality [12]. In all these studies, the prognostic impact of acute myocardial infarction complicated by CS was compared to that of patients without CS. A limited number of studies included CS patients only [15,16,26] and investigated their long-term mortality rate and predictors [15,16]. These studies reported a very high long-term mortality, up to almost 70% at six years suggesting that the risk of death persists in the years following the acute phase. Of note, they included patients with both STEMI and non-STEMI treated with different therapeutic strategies. Moreover, two of these were large randomized trials [15,16] with specific inclusion criteria potentially limiting the generalizability of their results. Thus, we aimed to assess the impact of CS on long-term mortality in a large, non-selected, real-world cohort of STEMI patients and developed a simple clinical score to predict their cumulative mortality.

**Figure 6:**
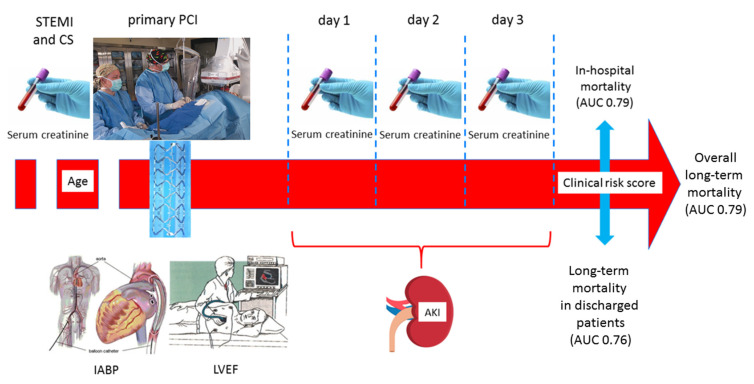
Schematic representation of the clinical score calculation in ST-segment elevation acute myocardial infarction (STEMI) patients with cardiogenic shock (CS) for in-hospital and long-term mortality prediction. AKI = acute kidney injury; AUC = area under the curve; IABP = intra-aortic balloon pump; LVEF = left ventricular ejection fraction; PCI = percutaneous coronary intervention.

In our study population, encompassing 465 STEMI patients, we observed an overall 60% mortality rate at long-term follow-up, including a 34% in-hospital mortality. Notably, the yearly mortality rate after discharge from the index hospitalization was 7%, with an overall 40% rate of long-term mortality observed in patients discharged alive from the index hospitalization. Moreover, long-term mortality remained unchanged throughout the 14-year period considered in our study. Thus, despite early coronary revascularization in all patients, mortality remained high, with about two thirds of discharged patients dying in the following years. These results prove that the adverse prognostic impact of CS is not limited to the acute phase but persists in the following years [15,16,26]. Therefore, additional intensified medical therapy and potential interventional strategies may be required in high-risk surviving patients. Of note, to meet this target an early and accurate stratification of risk is needed. We identified four simple clinical predictors of overall mortality that, when considered together, allowed an accurate risk discrimination during the first days of hospitalization. In particular, age, admission serum creatinine and LVEF, and development of AKI were independently associated with mortality. The close association between these variables and mortality in CS patients is not surprising, since cardiac and renal dysfunction and advanced age are known to be among the strongest predictors of death in STEMI [27,28,29]. Moreover, a close link between AKI and increased in-hospital mortality in patients with CS has been demonstrated in several studies [30]. In addition, our study confirms the prognostic relevance of AKI in CS patients also at long-term follow-up. This finding is in line with the results of the Intra-aortic Balloon Pump in Cardiogenic Shock (IABP-SHOCK) II trial [16], which is the only study evaluating clinical predictors of very long-term mortality in patients with CS in the current era. Indeed, Thiele et al. [16] found that oliguria, defined as urine output < 30 mL/h, which is an equivalent of severe AKI, is independently associated with 6-year mortality. However, differently from the Thiele et al. study that identified predictors of 6-year mortality and stratified patients into three risk categories (low, intermediate, and high) [23], our study is the first to integrate simple clinical predictors of long-term mortality into a risk score that allows physicians to accurately predict in-hospital and long-term mortality in each patient. Indeed, our risk score may have wide applicability and can be easily calculated in the initial days of hospital stay in order to help physicians select patients with a worse prognosis to be rapidly started on advanced mechanical circulatory support treatments and/or referred for transplant evaluation (Figure 6).

Notably, our risk score showed a good predictive accuracy for in-hospital mortality, with an AUC (0.79) similar to that observed for the six-variable score developed in the IABP-SHOCK II trial population for 30-day mortality [23]. A similar predictive accuracy was found when our score was applied to all-cause mortality following discharge from the index hospitalization (*p* = 0.11 between the two AUCs).

Some limitations of our study warrant mention. First, it is a retrospective study with the inherent limitations of such design including unmeasured confounders. Second, we evaluated a STEMI population treated in all cases with pPCI and IABP. As this therapeutic strategy may have influenced the study results, the overall applicability of our findings to patients with acute myocardial infarction complicated by CS, who do not undergo pPCI or treatment with other mechanical supports, needs to be clarified. Moreover, although the current clinical guidelines do not recommend routine use of IABP in patients with CS, our study reflects clinical practice during the study enrollment period. Notably, IABP still remains the most commonly used first-line support in this critical setting, as recently reported [31]. Furthermore, the extent of coronary artery disease and the promptness, completeness, and efficacy of myocardial revascularization were not assessed as confounders. In addition, different PCI techniques, coronary stents (bare-metal vs. drug-eluting stents), revascularization strategies (treatment of the culprit lesion only or multi-vessel revascularization during pPCI), and antithrombotic agents were used during the study period. Yet this corresponds to a “real-world” scenario where patients are treated with different antiplatelet drugs and stents according to operator choice, drug/device availability, and guideline recommendations. Moreover, no information was available regarding patients’ adherence to treatment during follow-up. Another limitation is that we collected information on long-term all-cause mortality only and not on cardiovascular mortality. Therefore, the accuracy of our score in predicting long-term cardiovascular mortality remains to be established. Finally, although we attempted to control for selection bias with cross-validation analysis, the lack of a validation cohort will require further confirmation on the clinical applicability of our score.

In conclusion, in STEMI patients with CS at hospital admission the risk of death is still substantial in the years following the index event. A simple clinical score applied during the first days of hospital stay can accurately predict early and late mortality risk. Future studies should validate this risk score and evaluate its usefulness in guiding the selection of patients in whom concentrate efforts in terms of early referral for mechanical circulatory support and heart transplantation.

## Figures and Tables

**Table 1:** Baseline characteristics and in-hospital complications of survivors and non-survivors at overall follow-up, including the in-hospital and the post-discharge observation periods.

Variable	Survivors (*n* = 184)	Nonsurvivors (*n* = 281)	*p* Value
Age (year)	63 ± 12	72 ± 11	<0.0001
Men, *n* (%)	144 (78%)	194 (69%)	0.03
Body weight (kg)	73 ± 13	71 ± 13	0.13
Hypertension, *n* (%)	90 (49%)	172 (61%)	0.01
Diabetes mellitus, *n* (%)	34 (18%)	63 (22%)	0.31
Smoking, *n* (%)	97 (53%)	128 (46%)	0.13
Dyslipidemia, *n* (%)	68 (37%)	97 (35%)	0.59
Anterior MI, *n* (%)	119 (65%)	178 (63%)	0.77
Prior MI, *n* (%)	27 (15%)	55 (20%)	0.17
Prior CABG, *n* (%)	6 (3%)	12 (4%)	0.58
LVEF (%)	37 ± 13	33 ± 12	0.003
Time-to-reperfusion (hours)	4.7 ± 3.8	5.2 ± 4.2	0.19
Serum creatinine (mg/dL)	1.15 ± 0.6	1.43 ± 0.8	<0.0001
eGFR (ml/min/1.73 m^2^)	73 ± 23	58 ± 23	<0.0001
Serum glycemia (mg/dL)	193 ± 94	226 ± 108	0.003
Contrast volume (mL)	235 ± 107	230 ± 116	0.64
Contrast ratio	0.73 ± 0.42	0.94 ± 0.78	0.002
Contrast ratio > 1, *n* (%)	21 (13%)	75 (27%)	0.001
New-onset AF, *n* (%)	29 (24%)	93 (34%)	0.003
VF before admission, *n* (%)	11 (6%)	14 (5%)	0.64
AKI, *n* (%)	26 (14%)	115 (41%)	<0.0001
Blood transfusions, *n* (%)	29 (16%)	61 (22%)	0.11

AF = atrial fibrillation; AKI = acute kidney injury; CABG = coronary artery bypass graft surgery; eGFR = estimated glomerular filtration rate (MDRD equation); LVEF = left ventricular ejection fraction; MI = myocardial infarction; VF = ventricular fibrillation.

**Table 2:** Logistic regression model of long-term mortality risk score.

Variables	*β* Coefficient
Age (years) (continuous)	0.0431
Left ventricular ejection fraction (%) (continuous)	−0.0359
Serum creatinine (mg/dL) (continuous)	0.2878
Acute kidney injury (yes vs. no)	0.6991
Constant *β*_0_	−3.6278

*β*_0_ is the constant of the logistic regression equation and *β* coefficient is the coefficient of each variable in the logistic regression equation, estimated at cross validation analysis.

**Table 3:** Characteristics of studies investigating the impact on long-term mortality of acute myocardial infarction complicated by cardiogenic shock.

First Author (Ref#)	Year of Publication	Study Acronym	Study Design	Study Population	Patient Treatment	Patients (*n*)	Follow-Up (years)	Mortality Rate (%)
Hochman [15]	2006	SHOCK	RCT	STEMI	Early PCI/CABG vs. medical stabilization	302	median 5.9	67% vs. 80%
Aissaoui [22]	2012	USIK 1995 USIC 2000 FAST-MI	Registries	STEMI/NSTEMI	PCI/TL/MT	486	1	82% (USIK) 67% (USIC) 76% (FAST-MI)
Spyridopolous [13]	2015	-	Registry	STEMI	pPCI	155	median 1.2	46%
Doost Hosseiny [12]	2016	-	Registry	STEMI	pPCI	92	mean 3.5	59%
Hemradj [11]	2016	-	Registry	STEMI	pPCI	387	1	30%
Kawaji [14]	2018	CREDO-KYOTO	Registry	STEMI	pPCI	466	5	51%
Thiele [16]	2018	IABP-SHOCK II	RCT	STEMI/NSTEMI	PCI vs. PCI + IABP	591	median 6.2	66% vs. 67%

CABG = coronary artery bypass graft; CREDO-KYOTO = Coronary Revascularization Demonstrating Outcome Study-Kyoto; FAST-MI = The French registry of Acute ST elevation or non-ST-elevation Myocardial Infarction; IABP = intra-aortic balloon pump; IABP-SHOCK = Intra-aortic Balloon Pump in Cardiogenic Shock; MT = medical therapy; NSTEMI = non-ST-elevation myocardial infarction; PCI = percutaneous coronary intervention; pPCI = primary percutaneous coronary intervention; RCT = randomized clinical trials; SHOCK = SHould We Emergently Revascularize Occluded Coronaries for Cardiogenic shocK; STEMI = ST-elevation myocardial infarction; TL = thrombolysis; USIC = Unité de Soins Intensifs Coronaires.

## Data Availability

Data and material will be available upon request.
